# Utility of Exogenous Ketones and Modified Atkins Diet in a Child With GSD VII (Tarui Disease)

**DOI:** 10.1002/jmd2.70123

**Published:** 2026-09-23

**Authors:** Kiera Batten, Nancy Van Doorn, Ji Hyun Moon, David Simar, Carolyn Broderick, Kaustuv Bhattacharya

**Affiliations:** ^1^ Department of Nutrition and Dietetics The Children's Hospital at Westmead Sydney Australia; ^2^ Genetic Metabolic Disorders Service, the Children's Hospital at Westmead Sydney Australia; ^3^ School of Health Sciences, Faculty of Medicine and Health, UNSW Sydney Sydney Australia; ^4^ Children's Hospital Institute of Sports Medicine, the Children's Hospital at Westmead Sydney Australia; ^5^ Heat and Health Research Centre, University of Sydney Sydney Australia; ^6^ School of Clinical Medicine, Faculty of Medicine and Health, UNSW Sydney Sydney Australia; ^7^ Trinity School of Medicine, Trinity College Dublin Dublin Ireland

**Keywords:** cardiopulmonary exercise test, glycogen storage disease, ketogenic, paediatric, phosphofructokinase

## Abstract

Glycogen storage disease VII (GSD VII), also known as Tarui disease, is caused by a deficiency of the skeletal muscle enzyme, phosphofructokinase (PFK). Classical GSD VII is characterised by reduced exercise capacity and exercise‐induced myopathic symptoms, exacerbated with carbohydrate intake. No recommended medical or dietary therapy currently exists to improve exercise symptomology in GSD VII. An 11‐year‐old female was recommended four trial dietary therapies for 8 weeks each: (1) usual diet, (2) 150 mg/kg exogenous ketones pre‐exercise, (3) modified Atkins diet (MAD), (4) MAD and pre‐exercise ketones. Clinical assessments comprised cardiopulmonary exercise test, anthropometry, biochemical tests including creatine kinase (CK), quality of life (QoL), heart rate (HR) monitoring and subjective symptom reports. Exogenous ketones improved exercise capacity demonstrated by reduced HR for equivalent exercise workload, and participation in higher intensity exercise for longer duration. MAD improved QoL physical domain score by 29%, normalised CK and halved the number of symptoms reported per ‘physically active day’. Dissatisfaction with MAD resulted in unintentional weight loss, and a 20% reduction in QoL emotional domain score, leading to the patient declining the fourth study phase. This study is the first to demonstrate a beneficial effect of exogenous ketones, and separately, MAD, in a child with GSD VII. Exogenous ketones or MAD could be recommended under medical and dietetic supervision in GSD VII. However, consideration must be given to the potential negative impact of MAD on weight and emotional health. Further interventional studies are warranted in this condition.

## Key Points

- No recommended medical or dietary therapy currently exists to improve exercise symptomology in GSD VII.
- This is the first study to demonstrate benefit of exogenous ketones, and modified atkins diet, in a child with GSD VII.
- Considerations should be given to the possible negative impact MAD may have on the emotional well‐being and growth in a child.

## Graphical abstract

A child with glycogen storage disease VII (Tarui disease) undertook three different diet therapies for 8 weeks each; usual diet, pre‐exercise exogenous ketones (150 mg/kg) and a modified atkins diet. Compared to usual diet, both exogenous ketones and modified atkins diet improved exercise‐induced myopathic symptoms, physical health‐related quality of life and exercise capacity. Modified atkins diet negatively impacted emotional health‐related quality of life, and also led to unintentional weight loss. This is the first study to demonstrate a beneficial effect of exogenous ketones, and separately, modified atkins diet, in a child with Tarui disease.

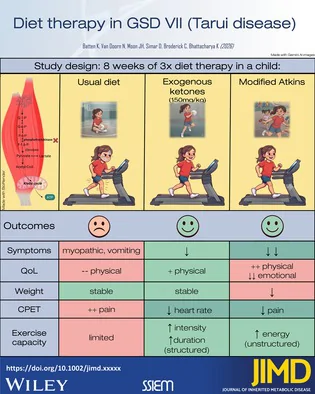

## 1 Introduction

Glycogen storage disease Type VII (GSD VII), also known as Tarui disease, results from deficiency of the enzyme phosphofructokinase (PFK), in skeletal muscle [1, 2]. GSD VII is a very rare inherited metabolic disorder (IMD), with only 100–200 published cases since first identified in 1965 [3, 4]. It is possible that prevalence of GSD VII may be underreported due to late or missed diagnosis, similar to McArdle disease [4]. Four phenotypes in GSD VII have been suggested; classical, late onset, severe infantile and haemolytic [3]. The classical and late‐onset forms appear to feature similar exercise‐induced myopathic symptoms to McArdle disease (GSD V), including pain, weakness, fatigue, vomiting and rhabdomyolysis [1, 2, 5, 6, 7, 8, 9, 10, 11, 12, 13, 14, 15, 16, 17, 18, 19]. Reduced exercise capacity has also been reported in GSD VII, with five studies totaling 16 adults indicating a 50% reduction in maximal oxygen uptake (VO_2_ peak) compared to healthy controls [19, 20, 21, 22, 23]. Despite limited available evidence, clinical practice guidelines recommend regular aerobic exercise to maintain or improve cardiorespiratory fitness [4].

Unlike McArdle disease, carbohydrate intake detrimentally impacts exercise capacity in GSD VII, termed an ‘out of wind’ phenomenon. This phenomenon has been clinically described in seven adults, where carbohydrate intake pre‐exercise resulted in increased heart rate (HR), decreased power output and reduced VO_2_peak, compared to exercise when fasted [10, 20, 21, 24]. Five other adult case studies describe a trial of either a modified Atkins diet (MAD), triheptanoin, or a high protein diet with branched chain amino acids, with the aim to improve exercise capacity [22, 23, 25, 26]. Only the MAD was reported to improve myopathic symptoms and exercise tolerance in a single adult [22]. One infantile case also described use of a classical ketogenic diet with subjective improvement in muscle strength and motor skills [25]. No studies have evaluated exogenous ketones in this cohort.

We describe here a case report of a child with GSD VII who undertook multiple clinical dietary therapies to assess impact on exercise capacity.

## 2 Presentation

This 11‐year‐old female reported experiencing exercise‐related symptoms from the age of 4–5 years, vomiting immediately after running more than 70 m. Taking up gymnastics at age 7, she learned to modify movements from continuous to ‘stop/start’, which reduced frequency of vomiting episodes. Netball was subsequently trialled with a similar approach and regular breaks. Parents report the patient was significantly physically fatigued after exercise, sometimes needing up to 4 h rest immediately following exercise, with full recovery taking up to 48 h. Vomiting was reported to be fast‐onset and not usually associated with nausea. Other symptoms described during or post‐exercise included sore stomach, sore legs and/or headache. Symptoms could arise during ad‐hoc physical activity (e.g., games during school lunch), or with structured exercise. At the age of 9, a neuromuscular gene panel identified heterozygous variants in the PFKM gene: c.116G>C p.(Arg39Pro) and c.1501–2del p.(?). The patient was subsequently referred to the paediatric IMD, and sport and exercise medicine services.

## 3 Methods

The clinical protocol was co‐designed by the treating team and family. The protocol was prospectively approved by the Sydney Children's Hospitals Network (SCHN) independent drug and therapeutics committee. Written, informed consent was subsequently given by the family, and the case report was approved by SCHN Ethics (CCR2026/6). Each therapy was planned for 8 weeks duration and included assessment of exercise capacity, dietary intake, anthropometry and quality of life (QoL); as well as HR and symptom monitoring.

### 3.1 Cardiopulmonary Exercise Test (CPET)

Sub‐maximal CPET was conducted on a treadmill (Medisoft Model 870A, MGC Diagnostics, USA), with continuous HR monitoring via a chest strap (Zephyr HXM, USA) [27]. Respiratory gases were collected using a mask (7450 V2, Hans Rudolph Inc., USA) and analysed breath by breath (Ultima System Cardio2 model 800 840‐021, MGC Diagnostics, USA). The modified (no slope) Bruce protocol was utilised, commencing at 2.7 km/h for 3 min, increasing in speed every 3 min until HR achieved 60%–70% of predicted HRmax using Tanaka formula (208‐[0.7*age]) [28, 29, 30, 31]. CPET was terminated after completion of the 3 min stage at target HR. Ratings of perceived exertion (RPE) and perceived pain (RPP) were taken at the end of every minute (modified Borg scale 1–10) [32].

### 3.2 Biochemistry

A cannula was inserted for blood draw 30 min pre‐exercise (range 20–40 min), and within 10 min post‐exercise. Biochemical tests included liver function tests (aspartate aminotransferase (AST), alanine aminotransferase (ALT), gamma‐glutamyl transferase (GGT), alkaline phosphatase (ALP)), ammonia, glucose, lactate, creatine kinase (CK), free fatty acids (FFA) and beta‐hydroxybutyrate (BHB).

### 3.3 Dietary Interventions

Therapy 1: Baseline: usual diet.

Therapy 2: Ketones: Exogenous ketone salts (generic product via hospital pharmacy: beta‐hydroxybutyrate +0.08% sodium acetate) prescribed 150 mg/kg, mixed with water and given 20 min pre‐structured exercise [33, 34, 35]. Frequency guided by current exercise participation, expected 2–3 per week.

Therapy 3: MAD: 3‐week transition onto diet, then target 10 g carbohydrate and 8–9 × 15 g fat exchanges daily, guided by a clinical dietitian.

Therapy 4: MAD + exogenous ketones as above.

The family supplied a 3‐day food diary sample for each therapy block, analysed by Foodworks 10 Professional (Xyris Software, Australia, 2019).

### 3.4 Quality of Life

PedsQL Pediatric QoL Inventory Generic Core Scales (approved by Mapi Research Trust) was completed at the end of each 8‐weeks, by both patient and proxy [36, 37, 38].

### 3.5 Home Monitoring

The patient wore a wrist HR monitor (FitBit Inspire 3, Google, USA) during waking hours. Parents observed HR data real‐time during structured sport, and retrospectively accessed HR data at the end of the day. Daily physical activity, exercise, ketone intake and any symptoms were recorded by the patient and parents. Urine ketone monitoring was recommended twice daily during MAD, target 4–8 mmol/L [39].

## 4 Results

### 4.1 Therapy Blocks

The patient completed each 8‐week intervention except for therapy 4. Due to the reported negative social, emotional and anthropological impact of MAD during therapy 3, the family and treating team decided not to undertake therapy 4.

### 4.2 CPET

**FIGURE 1:**
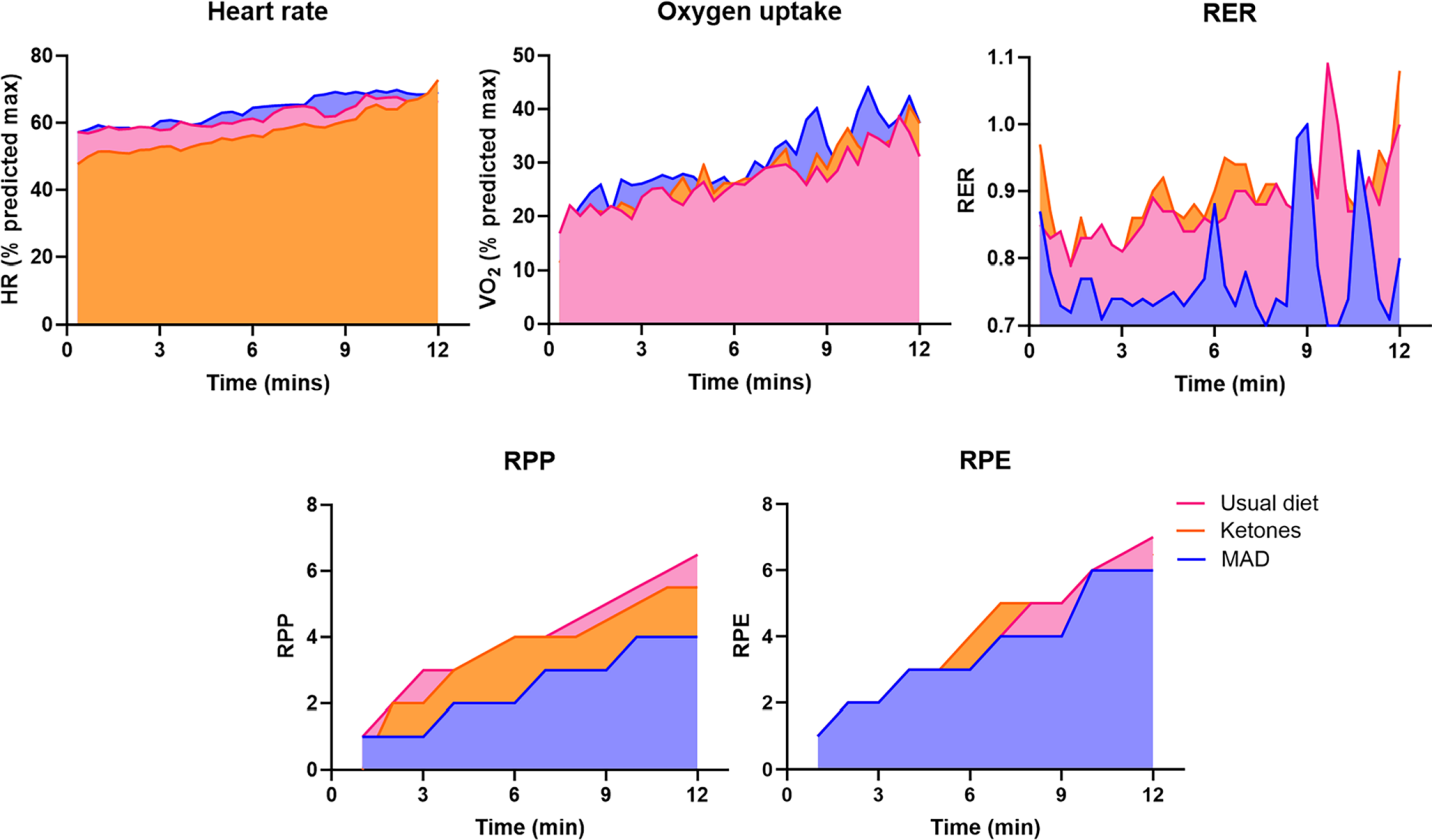
Cardiopulmonary exercise test (CPET) during each therapy block: Heart rate (HR), oxygen uptake (VO_2_), respiratory exchange ratio (RER), rating of perceived pain (RPP), rating of perceived exertion (RPE). Each 3‐min increment reflects a protocol stage.

Four stages (12 min) of the protocol were completed for all three interventions. The patient achieved 69% (range 66%–73%) of predicted HRmax, and 35% (range 31%–37%) of predicted VO_2_max (Wasserman formula) by the end of each protocol [40]. HR trended lower across almost all time points using ketones (see Figure 1). VO_2_ and HR trended higher on MAD compared with baseline or ketones. Respiratory Exchange Ratio (RER) and RPP trended lower on MAD, while RPE appeared similar across all therapies. Reported symptoms post‐CPET (sore stomach, sore head, fatigue) were subjectively most severe under baseline conditions, and least severe with MAD.

### 4.3 Biochemistry

BHB was elevated post CPET using ketones, and pre and post CPET with MAD. AST and CK were elevated pre and post CPET during baseline and ketone interventions, but not MAD. FFA was reduced post CPET using ketones, though remained in normal range. Ammonia, glucose, lactate, ALT, GGT and ALP did not differ between dietary interventions or pre and post CPET (see Table S1).

### 4.4 Anthropometry

**FIGURE 2:**
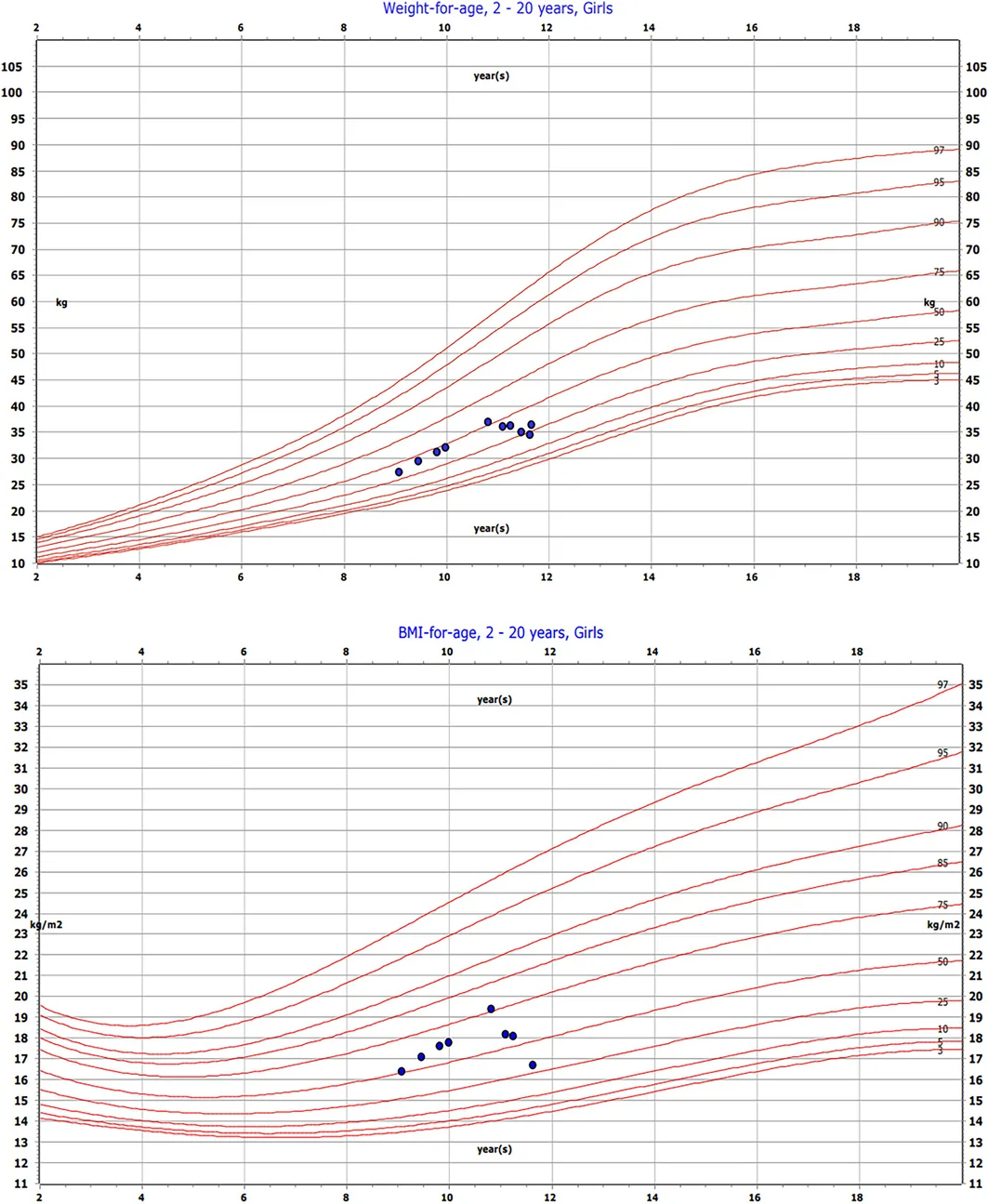
Weight for age (top) and Body Mass Index (BMI) for age (bottom). Noting clinical therapy blocks commenced at age 11, with the final weight plotted after transitioning off MAD.

Weight and BMI Z‐scores reduced from baseline to end of MAD (see Figure 2). Height Z‐score remained stable across all interventions.

### 4.5 Food Intake

Estimated energy and protein requirements were determined as 1800 kcal/day and 32 g/day, respectively [41, 42]. Proportion of fat and carbohydrate intake were similar during baseline and ketone therapies, providing 40% and 45% energy intake (EI) respectively. During MAD, this changed to 70% EI from fat and 5% EI from carbohydrate (see Table S2). The patient reported finding the ketone salts unpalatable and mitigated this by adding an unspecified volume of artificially sweetened cordial. Both the patient and parents reported finding the MAD very challenging, experiencing repeated constipation, being ‘mentally and emotionally draining’, and ‘sad about missing out [on foods others were eating]’. Parents reported the patient became less interested in food and inadvertently reduced overall caloric intake during therapy 3. Though samples were offered, no specialised medical food or formula was utilised by the patient during MAD.

### 4.6 Quality of Life

**FIGURE 3:**
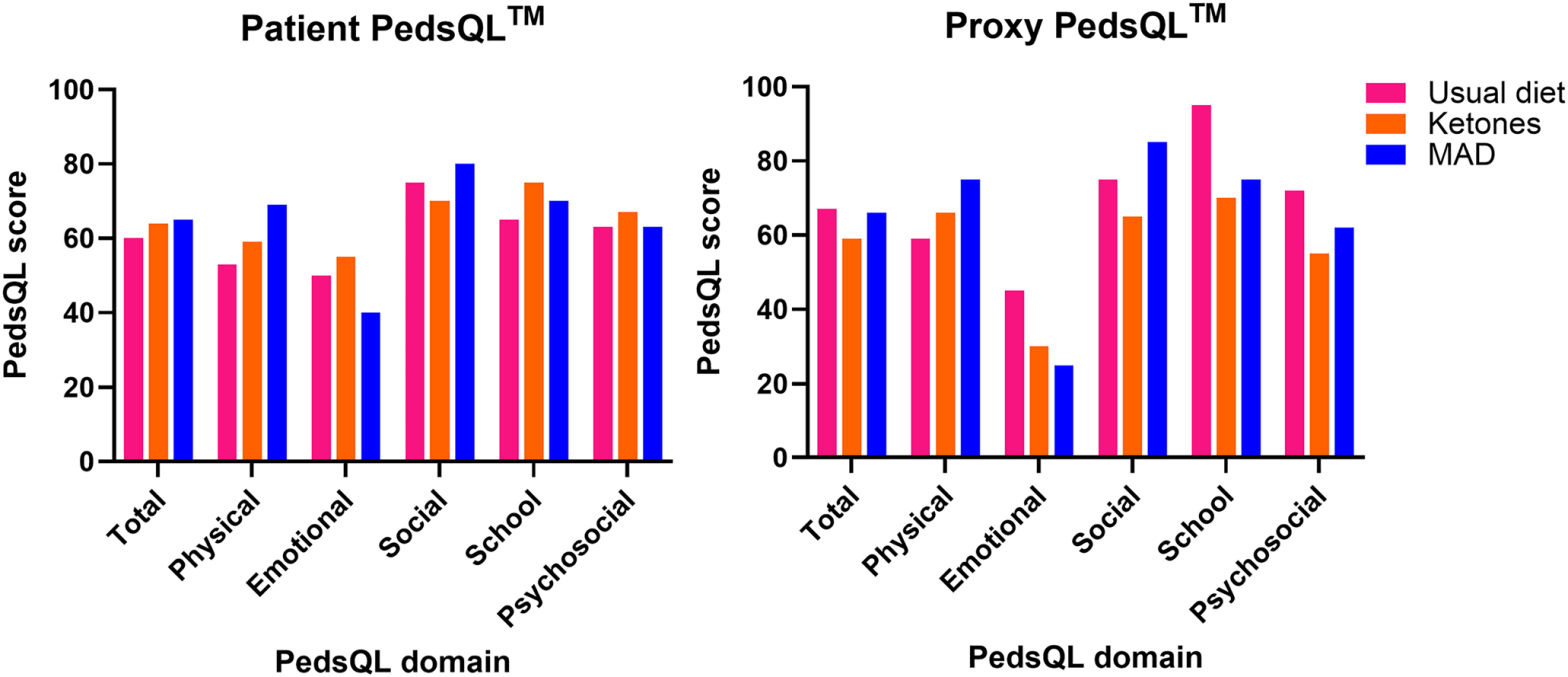
PedsQL Pediatric Quality of Life Inventory Generic Core Scales for each therapy block for patient and proxy (parent). Higher scores infer better health‐related quality of life.

Compared to baseline, PedsQL indicated improved physical domain scores with MAD, by 29% and 26% for self and proxy respectively; and with ketones, by 12% and 11% for self and proxy respectively. Emotional domain score was reduced with MAD, by 20% and 44% by patient and proxy, respectively. Total PedsQL score appeared similar across therapies (see Figure 3).

### 4.7 Home Monitoring

The family recorded 57 days (range 55–61) per therapy block. Symptoms included: stomach pain, headache, sore legs, nausea, vomiting, out of breath, tired, dizzy and/or shaky. The number of symptoms reported per ‘physically active day’ reduced from 2.5 and 2.4 during baseline and ketone therapies respectively, to 1.2 during MAD (see Table S3).

The family reported an overall increased tolerance to netball duration from 5–10 min per quarter for 2–3 quarters at baseline, to 10 min per quarter for 4 quarters with ketones. Parents also noted symptoms occurred when HR exceeded 70% predicted maximum at baseline; but with ketones, 20 min of HR at 80%–90% predicted maximum was tolerated. The family observed ketones resulted in minimal or considerably reduced symptoms during and after structured exercise. However, symptoms continued during unstructured physical activity when ketones were not consumed. Conversely, MAD resulted in very mild or no symptoms during unstructured physical activity. During MAD, the parents observed an overall improvement in energy levels, and on one occasion, the patient noted she ran ‘faster than ever before’. Parents also noted that some symptoms recorded during MAD were likely attributed to hunger, rather than exercise.

Home measured urinary ketones during MAD were reported on 42 occasions, with a median value of 4 mmol/L.

## 5 Discussion

In this case report we describe the first clinical study evaluating multiple dietary interventions, in a child with GSD VII. We demonstrated a beneficial effect of exogenous ketones and separately, MAD, for improving exercise capacity and myopathic symptoms.

Elevated BHB post CPET indicated that 150 mg/kg of ketone salts was adequate to achieve therapeutic ketosis. Similarly, MAD resulted in elevated blood ketones and consistent urinary levels to target range, indicating ketosis was achieved and ketone bodies were available as a fuel source for the working muscle.

At baseline, our patient experienced similar exercise‐induced symptoms to other GSD VII cases [4]. Home symptom reporting indicated ketones improved exercise‐induced symptoms and exercise capacity during structured physical activity, and MAD during ad‐hoc physical activity. Due to school holidays, the patient did not participate in netball during MAD. Thus, a comparison to baseline and ketones was not possible. Correspondingly, the number of ‘physically active days’ was lowest during MAD, which may impact the number of reported symptoms and may limit interpretation of results. Nonetheless, the number of symptoms per ‘physically active day’ halved during MAD compared to baseline or ketones, indicating a relative reduction in exercise‐induced symptoms. Equally, the patient observed an increase in symptoms after transitioning off MAD.

CK and AST normalised during MAD, indicating reduced muscle breakdown, while PedsQL indicated improved physical QoL. [4] However, again the lack of higher‐intensity structured exercise during MAD may confound these results.

We utilised sub‐maximal CPET, so cannot compare our VO_2_peak values to maximal CPET normative data, which has been used to describe reduced aerobic capacity in other GSD VII cases [19, 20, 21, 22, 23]. During sub‐maximal CPET, ketones resulted in lower HR, but comparable VO_2_ for the same workload compared to baseline. As VO_2_ is a product of cardiac output and arterial–venous oxygen difference (a‐VO_2_), this would infer ketones improved oxygen uptake and utilisation by skeletal muscle, or increased stroke volume [40, 43]. Reduced oxygenation of skeletal muscle has been observed using near infrared spectroscopy in three adults with GSD VII, and reduced a‐VO_2_ peak has been described in seven adults with GSD VII [10, 20]. There are no published reports on stroke volume in this population. Ketones also appeared to improve exercise capacity at higher intensities. This was indicated by an absence of symptoms at sustained higher %HR maximum during netball using ketones, compared to presence of symptoms at lower %HR maximum of shorter duration, without ketones.

MAD demonstrated the lowest RPP on CPET, inferring reduced myopathic symptoms, which corresponded to a reduction in home‐reported symptoms during MAD therapy. MAD appeared to result in the highest HR and VO_2_ compared to baseline and ketones at equivalent workload. If a‐VO_2_ was equivalent using ketones and MAD, the higher VO_2_ would be directly attributed to the higher HR. The higher HR appears surprising when compared to the HR response with exogenous ketones. As HR can be affected by additional factors such as heat, fatigue, stress; and we did not measure stroke volume, we are unable to delineate these results further [44]. In addition the impact of GSD VII in cardiac tissue PFK isoenzymes is still unknown [45]. Predictably, RER was lowest during MAD, reflecting higher fat oxidation [40].

No case studies have investigated use of exogenous ketones in GSD VII. Only one adult case report describes evaluation of MAD in GSD VII using CPET, indicating a minor improvement to workload, relative VO_2_peak and myopathic symptoms [22]. Unlike the adult case, however, our patient did not tolerate MAD well. MAD had a clear detrimental impact on her emotional, mental and social well‐being, as indicated via PedsQL, loss of weight and observations from the family including feelings of sadness, guilt and being left out.

In discussion with the treating team and family, the family ultimately opted for a ‘lower carbohydrate diet’ for ongoing management, aiming for 30–40 g carbohydrate per day, but without restricting foods the patient enjoyed. Ketones were recommended 20 min pre‐structured exercise at 150 mg/kg. Ketone use before unstructured exercise, such as school recess or lunch, could also be considered. Home HR monitoring during exercise could be useful in mitigating symptoms during structured exercise, aiming to avoid prolonged periods over 70%–80% HR maximum.

## 6 Conclusion

This is the first case report of a paediatric patient with GSD VII to evaluate the effect of pre‐exercise ketones and MAD on exercise capacity and myopathic symptoms. Although both diet therapies were found to be beneficial, the negative emotional impact of MAD was determined not to be in the patient's best interest for long‐term clinical therapy. Future consideration should be given to optimal ketone dose and frequency, and/or a less restrictive lower carbohydrate diet in larger cohort studies in GSD VII.

## Author Contributions

Kaustuv Bhattacharya, Kiera Batten, Carolyn Broderick and Nancy Van Doorn conceptualised the clinical study and outcome analysis. Kiera Batten, Kaustuv Bhattacharya, Ji Hyun Moon, Nancy Van Doorn and Carolyn Broderick were part of the clinical treating team and collected clinical and exercise data. Kiera Batten, Kaustuv Bhattacharya, David Simar and Ji Hyun Moon undertook data analysis and interpretation. Kiera Batten drafted the manuscript. All contributors revised, edited and approved the final manuscript.

## Funding

Funding was received by the Sydney Children's Hospital Foundation. K.B. received a tuition fee offset and a stipend from UNSW through the Australian Government Research Training Program. The authors confirm independence from the sponsors; the content of the article has not been influenced by the sponsors.

## Disclosure

There has been no use of AI in the production of this manuscript except for the graphical abstract (Gemini AI).

## Ethics Statement

Ethics approval was obtained from the Sydney Children's Hospital Network Human Research Ethics Committee (CCR2026/6).

## Consent

Patient and guardian written consent was obtained from the patient and family prior to ethics submission and case report.

## Conflicts of Interest

The authors declare no conflicts of interest.

## Supporting information


**Table S1:** Blood analytes measured before (pre) and after (post) CPET, for each dietary intervention. ‘H’ indicates exceeds reference range.
**Table S2:** 3‐day food diary analysis. %EI = % of total energy intake.
**Table S3:** Home symptom monitoring.

## Data Availability

The data that support the findings of this study are available from the corresponding author upon reasonable request.
