# Supplementary material for: Utility of Exogenous Ketones and Modified Atkins Diet in a Child With GSD VII (Tarui Disease)
Source: JIMD Rep. 2026 Sep 23;67(5):e70123. doi: 10.1002/jmd2.70123 (PMC13599733; doi:10.1002/jmd2.70123)
Supplement: Supplementary file 1 [file JMD2-67-e70123-s001.docx]

**Supplementary Material**

*Table 1: Blood analytes measured before (pre) and after (post) CPET, for each dietary intervention. ‘H’ indicates exceeds reference range.*

| **Analyte** | **Reference** | **Baseline** | | **Ketones** | | **MAD** | |
| --- | --- | --- | --- | --- | --- | --- | --- |
|  |  | **Pre** | **Post** | **Pre** | **Post** | **Pre** | **post** |
| Aspartate aminotransferase (U/L) | 0-33 | 46 H | 44 H | 57 H | 58 H | 32 | 32 |
| Alanine aminotransferase (U/L) | 0-40 | 30 | 28 | 22 | 21 | 16 | 16 |
| Gamma-glutamyl transferase (U/L) | 0-45 | 17 | 18 | 13 | 13 | 17 | 15 |
| Alkaline phosphatase (U/L) | 100-460 | 136 | 133 | 170 | 170 | 143 | 145 |
| Glucose (mmol/L) | 3.5-7.7 | 4.7 | 4.7 | 4.7 | 4.4 | 4.4 | 4.6 |
| Ammonia (umol/L) | 0-50 | 33 | 37 | 34 | 36 | 20 | 32 |
| Lactate (mmol/L) | 0-1.9 | 1.4 | 1.1 | 1.0 | 0.8 | 1.7 | 1.3 |
| Creatine Kinase (U/L) | 15-180 | 473 H | 458 H | 1531 H | 1489 H | 119 | 117 |
| Cholesterol (mmol/L) | 0-5.1 | 5.2 H | 5.0 | 4.7 | 4.6 | 5.3* H | 5.1 |
| Triglycerides (mmol/L) | 0-1.5 | 0.9 | 1.0 | 0.8 | 0.8 | 1.6* H | 1.2 |
| Free fatty acids (umol/L) | 150-950 | 484 | 615 | 410 | 161 | 533 | 612 |
| Beta-hydroxybutyrate (mmol/L) | 0-0.5 | 0.29 | 0.27 | < 0.18 | 0.52 H | 0.89 H | 0.83 H |

Note 1 – all ‘pre’ samples taken at least 3 hours post food; may falsely elevate cholesterol and triglyceride values.

Note 2 – each CPET was conducted at the end of each 8-week dietary intervention, except for Ketones which was conducted at the start of the intervention block due to a miscommunication. Thus the higher CK observed pre/post CPET with Ketones is not comparable to the other therapies. As CK can take up to a week or more to return to baseline post muscle injury, the result observed pre/post ketone CPET may be falsely impacted by the preceding Baseline block.^(1,2)^

*Table 2: 3-day food diary analysis. %EI = % of total energy intake.*

|  | **Baseline** | **Ketones** | **MAD** |
| --- | --- | --- | --- |
| **Energy** (% kcal of estimated daily requirement) | 80% | 61% | 97% |
| **Protein** (% EI) | 14 | 16 | 24 |
| **Carbohydrate** (% EI) | 47 | 44 | 5 |
| **Fat** (% EI) | 37 | 37 | 70 |

Note 1 - there is likely underreporting of intake during Ketones, and overreporting of intake during MAD, in the context of subsequent loss of weight.

Note 2 – dietary intake during Baseline and Ketones was a variety of foods from all food groups, comparable to healthy peers.

*Table 3: Home symptom monitoring:*

|  | **# days recorded** | **% active days** | **% symptomatic days** | **# of symptoms reported per active day** |
| --- | --- | --- | --- | --- |
| **Baseline** | 55 | 80 | 68 | 2.5 |
| **Ketones** | 55 | 75 | 80 | 2.4 |
| **MAD** | 61 | 54 | 73 | 1.2 |

*List of abbreviations in the manuscript:*

PFK = phosphofructokinase

MAD = modified Atkins diet

CK = creatine kinase

QoL = quality of life

HR = heart rate

GSD VII = glycogen storage disease type VII

IMD = inherited metabolic disorder

VO_2_peak = maximal oxygen uptake

SCHN = Sydney Children’s Hospital Network

CPET = cardiopulmonary exercise test

RPE = rating of perceived exertion

RPP = rating of perceived pain

AST = aspartate aminotransferase

ALT = alanine aminotransferase

GGT = gamma-glutamyl transferase

ALP = alkaline phosphatase

FFA = free fatty acids

BHB = beta-hydroxybutyrate

PedsQL^TM^ = Pediatric QoL Inventory Generic Core Scales

RER = respiratory exchange ratio

BMI = body mass index

%EI = percent of energy intake

**References**

1.	Lucia A, Martinuzzi A, Nogales-Gadea G, Quinlivan R, Reason S (2021) Clinical practice guidelines for glycogen storage disease V & VII (McArdle disease and Tarui disease) from an international study group. *Neuromuscular Disorders* 31 (12): 1296–1310. Available from: 10.1016/j.nmd.2021.10.006.

2.	Shakerdi LA (2026) Metabolic myopathies and hyperCKemia in adulthood: a clinical approach to diagnosis and management. *Journal of Clinical Medicine* 15 (5).
